# Novel Gallium(III), Germanium(IV), and Hafnium(IV) Folate Complexes and Their Spectroscopic, Thermal Decomposition, Morphological, and Biological Characteristics

**DOI:** 10.1155/2020/6678688

**Published:** 2020-12-18

**Authors:** Abeer A. El-Habeeb

**Affiliations:** Department of Chemistry, College of Science, Princess Nourah Bint Abdulrahman University, Riyadh 11671, Saudi Arabia

## Abstract

In this study, we describe novel gallium(III), germanium(IV), and hafnium(IV) folate complexes, including their synthesis and analyses. The synthesized folate complexes were also subject to thermal analysis (TGA) to better examine their thermal degradation and kinetic properties. The folate complexes had high stability and were nonspontaneous. The Coats–Redfern and Horowitz–Metzger equations were used to determine thermodynamic parameters and describe the kinetic properties. These complexes were synthesized through the chemical interactions in neutralized media between the folic acid drug ligand (FAH_2_) with GaCl_3_, GeCl_4_, and HfCl_4_ metal salts at 1 : 2 (metal : ligand) molar ratio. The conductance measurements have low values due to their nonelectrolytic behavior. The X-ray powder diffraction solid powder pattern revealed a semicrystalline nature. In vitro, we screened the synthesized folate chelates for antibacterial and antifungal activities. The inhibition of four bacterial and two fungi pathogens (*E. coli*, *B. subtilis*, *P. aeruginosa*, *S. aureus*, *A. flavus*, and *Candida albicans*) was improved using a folic acid drug relative to the control drug.

## 1. Introduction

**Figure 1:**
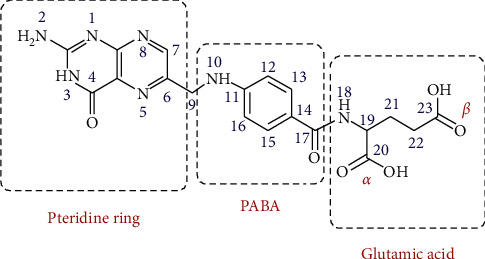
Structure of folic acid (FAH_2_).

Folic acid (FAH_2_) is also referred to as pteroylglutamic acid [*N*-(4{[(2-amino-4-oxo-1,4-dihydropteridin-6-yl)methyl]amino}benzoyl)-L-glutamic acid]. Folic acid is a member of the B9 vitamin family [1]. Folic acid consists of three sections of *p*-aminobenzoic acid (PABA) pteridine ring and glutamic acid moieties (Figure 1). The term folate refers to the deprotonated form of folic acid, which plays an essential role in critical biosynthetic processes in mammalian cells [2]. The folate molecule is made as a coenzyme in the case of DNA, RNA, and protein components synthesized by single carbon transfer reactions [3]. Folate is necessary for the synthesis of methionine, methylation of DNA, histone neurotransmitters, and lipids. The deficiency in folic acid led to DNA strand breaks [4], DNA hypomethylation [5], and abnormal gene expression [6]. Folic acid has a potential anticancer activity in some important cases, such as breast, colorectal, and ovarian carcinomas [7]. The World Health Organization has added folic acid to the list of trusted drugs that are most needed. For humans, this vitamin should be obtained from the diet because the human body cannot produce it. The recommended daily folate intake for adults is 400 *μ*g [8]. Folic acid is one of the major molecules for the synthesis of DNA [9]. Folic acid receptors (FRs) have been considered a target to assimilate the organic molecules associated with folic acid in cells. Application of folic acid receptor-targeted molecules is not new; there have been many articles addressing the conjugation of folates to known organic drug molecules, such as taxol, paclitaxel, and doxorubicin, to improve drug targeting [10–13].

However, there is a lack of literature concerning the conjugation of folic acid to metal-based molecules and even less regarding their chemotherapeutic potential. Incorporating simple folate ligands into biologically active metal complex systems might offer a simple strategy for improving their selective uptake in FR cells [2]; on the contrary, it was found that the metal complexes of folic acid might provide an advantage by increasing the inhibitory activity of folic acid [7]. Folic acid can form complexes with different metal ions via the pteridine ring or glutamic acid moiety. The pteridine ring binds to metal ions via its nitrogen and oxygen atoms (of a carbonyl or an iminol group) in the coordination process [14]. But this case is not common since the pteridine ring is a highly *π*-electron-deficient heterocycle, while glutamic acid forms stable complexes with metal ions [15]. Three types of coordination modes have been mentioned in literature on folic acid: bidentate bridging via *α* and *γ* carboxylate groups [16–20], tridentate (*α* and *γ* carboxylate groups) and the amide nitrogen of the glutamate moiety [2,21], and chelating via *α* carboxylate and the amide nitrogen of the glutamate moiety [22]. Many publications focus on the synthesis and characterization of metal-folate complexes. Still, they do not discuss their biological activity although such complexes might have high selectivity towards FR over expressing cells and thus a distinct biological activity and anticancer properties.

Here, we describe the synthesis of new metal-folate complexes as therapeutic agents. New metal-folate complexes were formed from the chemical reactions between FAH_2_ and some metal ions like Ga(III), Ge(IV), and Hf(IV). The synthesized folate complexes were discussed by using microanalytical, spectral tools, and thermogravimetric analysis. An antimicrobial assessment for the synthesized folate complexes towards bacteria and fungi species and the anticancer properties have been studied, for instance, on the germanium(IV)-folate complex against the human hepatocellular carcinoma (HepG-2) cell line.

## 2. Materials and Methods

### 2.1. Chemicals and Equipment

The pure grade chemical materials (GaCl_3_, GeCl_4_, HfCl_4_, and folic acid) were received from Sigma-Aldrich chemical company. The microanalytical, physical, and spectral measurements with corresponding models are listed as follows:

**Table:** 

Analysis technique	Instruments
Elemental analysis	PerkinElmer, CHN 2400
Conductance	Jenway 4010 conductivity meter
FTIR spectra	Bruker FTIR spectrophotometer
Electronic spectra	UV2 Unicam UV/Vis spectrophotometer
Magnetic susceptibility	Magnetic susceptibility balance
^1^HNMR spectra	Varian mercury VX-300 NMR spectrometer, 300 MHz
Thermogravimetric	TG/DTG–50H, Shimadzu thermogravimetric analyzer
SEM	Quanta FEG 250 equipment
XRD	X‘Pert PRO PAN analytical, with copper target
TEM	JEOL 100 s microscopy

### 2.2. Preparation of Folate Complexes

The folate complexes with the molecular formulas NH_4_[Ga(FA)_2_]·4H_2_O (1), [Ge(FA)_2_]·3H_2_O (2), and [Hf(FA)_2_]·3H_2_O (3) were synthesized using the following procedure. Mixtures of 2.0 mmol of GaCl_3_, GeCl_4_, or HfCl_4_ with 4.0 mmol of FAH_2_ were stirred in 25 mL of CH_3_OH solvent. The pH of the mixtures was adjusted to about 7.7–8.0 by adding ammonia solution (0.2M) dropwise and refluxed at about ∼60°C for 3 h. The solutions of these mixtures were reduced by leaving them still for 1 week. The solid yields of folate complexes reached 60–66%, with a higher melting point above 300°C. The microanalysis (%) of the elements (C, H, and N) for the three synthesized complexes is summarized as follows:

**Table:** 

Complexes	Elements	Calc. (%)	Found (%)
1	C	43.91	43.66
	H	4.27	4.20
	N	18.22	18.77

2	C	45.35	45.31
	H	4.26	4.21
	N	19.02	19.33

3	C	41.03	41.11
	H	4.36	4.31
	N	17.75	17.31

### 2.3. Antimicrobial Inhibitions

A modified Kirby–Bauer experiment was used to assess antimicrobial inhibitions [23]. A cytotoxicity assay for the germanium(IV)-folate complex was performed using the human hepatocellular carcinoma (HepG-2) cell line [24, 25]. The HepG-2 cell line was obtained from the VACSERA Tissue Culture Unit.

## 3. Results and Discussion

### 3.1. Molar Conductance

The molar ratios and conductance behaviors of gallium(III), germanium(IV), and hafnium(IV) folate complexes have been discussed. The elemental analyses are consistent with 1 : 2 (metal : ligand) stoichiometry. The synthesized complexes are insoluble in polar solvents but soluble in common organic solvents like DMF and DMSO. Gallium(III), germanium(IV), and hafnium(IV) folate complex solutions dissolved in DMSO showed slightly low conductance (Λ_m_ = 14–30 ohm^−1^ cm^2^ mol^−1^), suggesting a nonelectrolyte behavior [26].

### 3.2. Infrared Interpretations

**Figure 2:**
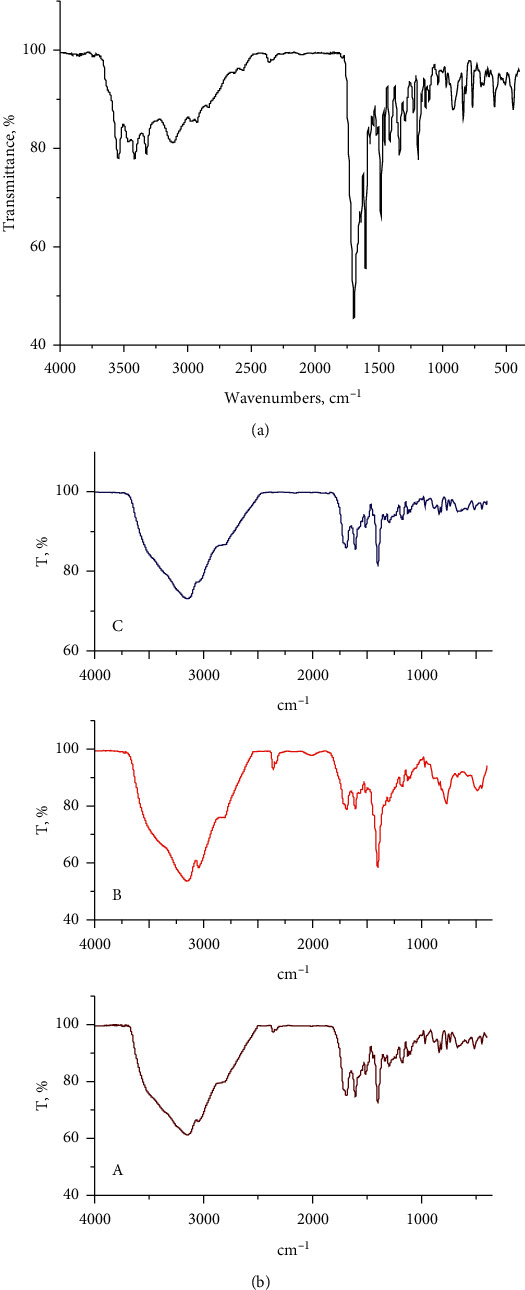
(a) Infrared spectrum of a pure folic acid drug; (b) infrared spectra of A : Ga(III), B : Ge(IV), and C : Hf(IV) folate complexes.

The FTIR spectra for the FAH_2_-free chelate and the synthesized metal complexes are shown in Figures 2(a) and 2(b). All complexes have similar spectra, which reflect similar structural characteristics for these complexes. The significant infrared spectral bands were assigned and inserted in Table 1. These assignments can be summarized with the following evidences:

- The folic acid-free ligand has a stretching vibration band at 1702 cm^*−*1^, which is attributed to *ν* (C*=*O)_ketonic_ of the carboxylic group; this band is overlapped with the *ν* (C=O) amide group [16]. This band was shifted to lower wavenumber in all complexes, with a marked decrease in the spectral intensity. Interestingly, the bands present at 1604 and 1485 cm^−1^ were assigned to *ν*_as_ (COO^−^) and *ν*_s_ (COO^−^) stretching vibrations in the case of the spectrum of the folic acid ligand. These bands were shifted to higher and lower frequencies, respectively, under complexation because of the involvement of the oxygen of the carboxylate group in the coordination towards metal ions. The difference between antisymmetric (*ν*_as_COO) and symmetric (*ν*_s_COO) stretching vibrations for the COO group gives an impression about the speculated molecular structure of the folate complexes [27]. The coordination mode of the carboxylate group was discussed by Deacon and Phillips [28] according to the Δ*ν* = [*ν*_as_ (COO)–*ν*_s_ (COO)] relationship. The interactions between the metal ions and the carboxylate group were (a) monodentate fashion when Δ*ν* >200 cm^−1^, (b) bidentate/chelating fashion when Δ*ν* is smaller than ionic form, and (c) bridging bidentate when Δ*ν* has nearly ionic values. The observed Δ*ν* values for all the complexes exhibited within the 211–217 cm^−1^ range, as discussed in Table 2. Therefore, the carboxylate groups chelated to metal ions as a unidentate mode [28].
- The FAH_2_-free ligand has a distinguished band at 3318 cm^−1^ that is attributed to the *ν* (N−H) of the amido group. This band blue-shifted in the case of the spectra of the synthesized complexes, and the characteristic band for *δ* (NH) amide is downshifted in the complexes relative to the free ligand. This result is in agreement with the HNMR data, which confirmed the participation of the amide nitrogen in the coordination of metal ions. So, this situation confirms the presence of a folate ligand as a tridentate ONO chelate through *α*-COO, *β*-COO, and amide groups [2, 21].
- There are two vibration bands in the case of the spectrum of FAH_2_-free ligand located at 3558 and 3411 cm^−1^, which are assigned to the *ν* (O–H) vibrations of the carboxylic groups. These bands are present with broadening in the spectra of folate complexes due to the presence of the crystalline water molecules [1, 29, 30].
- The weak and very weak intensity bands in the range of 600–400 cm^−1^ are attributed to stretching vibrations of M–N and M–O [31]. These data assume a tridentate manner of coordination for the folate chelate towards metal ions by deprotonating the two carboxylic groups of glutamic acid and NH of the amide group.

### 3.3. Electronic Spectra

**Figure 3:**
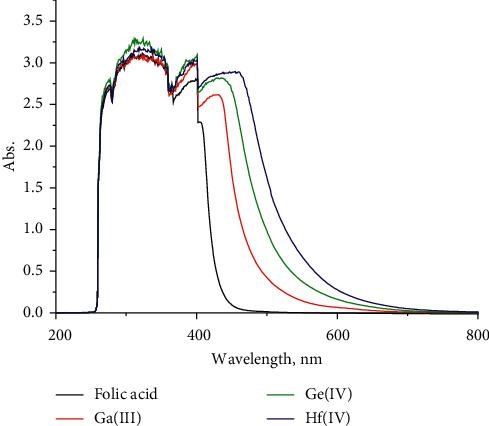
Electronic spectra of folic acid-free chelate and its Ga(III), Ge(IV), and Hf(IV) complexes.

The UV-visible spectrum of folic acid (Figure 3) exhibits absorbance bands at 220 and (290 and 380 nm) attributed to *π* ⟶ *π*^*∗*^ and *n* ⟶ *π*^*∗*^ transitions. The first peak is probably due to the alkyl and aromatic species, while the other bands are assigned to COOH, NH, NH_2_, and C=O groups [32]. These bands are shifted to longer wavelengths, which support the complexity of metal ions with carboxylic and amide groups (Figure 3). The folate complexes have bands within the 428–454 nm range due to the L ⟶ M_CT_ transitions [33, 34].

### 3.4. ^1^HNMR Study

The ^1^HNMR spectrum of the FAH_2_-free ligand in DMSO-d_6_ (Figure 1S and Table 3) has a quadrature signal obtained at *δ* 2.317 and 2.293 ppm of the protons H (21) and triplet signal at *δ* 2.504, 2.501, and 2.495 ppm of the protons H (22) due to the methylene CH_2_ group. The signals were shifted downfield in the case of the synthesized folate complexes (Figures 2S and 3S and Table 3). A signal at *δ* 8.093 ppm of the proton H (18) is due to the NH amide group. After complexation, this signal was upfield shifted with a chemical shift difference at 0.305–0.338 ppm, thus supporting the amido group's involvement in the chelation process [35]. The triplet signal at *δ* 4.476 ppm of the proton H (19) is due to the methylene group. This group significantly affected the spectra of the folate complexes, which was shifted to the low field. This was because of the effect of attaching the amide and carboxylic groups. A signal at *δ* 4.496 ppm of the proton H (9) is due to shifting of the methylene CH_2_ group to a low field, a result of the cycling effect from one side and the NH group effect from the other side. A single signal at *δ* 8.5 ppm of the proton H (7) is due to the (CH=N) 2-pyrazine group. These findings strongly support the coordination site through the tridentate fashion of the folate ligand [21, 35].

### 3.5. Thermogravimetric Analysis

**Figure 4:**
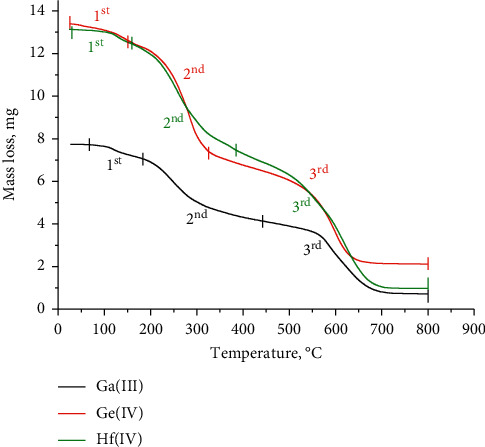
TGA curves of Ga(III), Ge(IV), and Hf(IV) folate complexes.

The stabilities of the thermal decompositions of the synthesized metal chelates were discussed based on the TGA analysis (Figure 4 and Table 4). The thermal decomposition of the NH_4_[Ga(FA)_2_]·4H_2_O complex (**I**) takes place through three degradation stages with a mass loss of 8.66% (temperature range between 56 and 181°C), 37.98% (temperature range between 181 and 445°C), and 44.056% (temperature range between 445 and 800°C), respectively. The remaining residual mass is due to the gallium metal contaminated with few unoxidized carbon atoms. The thermal decomposition of the [Ge(FA)_2_]·3H_2_O complex(**II**) passes through three steps. The first decomposition step is within the 30–146°C temperature range and has a mass loss of 5.45%. The second step is within the 146–321°C range and has a mass loss of 38.54%. The third step is within the 321–800°C range, with a mass loss of 39.58%. The germanium metal polluted with few unoxidized carbon atoms is a final residue at 800°C. The [Hf(FA)_2_]·3H_2_O complex(**III**) was decomposed through three steps within temperature ranges at 34–154°C, 154–380°C, and 358–800°C with mass losses of 4.86%, 33.55%, and 45.60%, respectively. At 800°C, the hafnium metal was mixed with some nonoxidized carbon atoms, which represents the residual material.

### 3.6. Kinetic and Thermodynamic Parameters

Using official integral methods, including Horowitz–Metzger (HM) [36] and Coats–Redfern (CR) methods [37], the parameters of the kinetic thermodynamic process are calculated and listed in Table 5. From the theoretical data, it can be deduced that the activation energies and, by extension, the thermal stability of the folate complexes are ordered as Ga(III) > Ge(IV) > Hf(IV). The results of the two methods used to calculate the thermodynamic parameters are satisfactorily consistent with each other. The pyrolysis steps of the folate complexes are nonspontaneous (∆S ^*∗*^) with negative data due to the thermal stability of the synthesized complexes.

### 3.7. Speculated Structure of Folate Complexes

**Figure 5:**
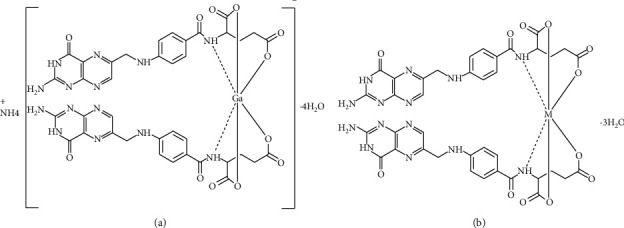
(a) Speculated structure of the Ga(III) folate complex; (b) speculated structures of Ge(IV) and Hf(IV) folate complexes.

The proposed structures of NH_4_[Ga(FA)_2_]·4H_2_O (**1**), [Ge(FA)_2_]·3H_2_O (**2**), and [Hf(FA)_2_]·3H_2_O (**3**) folate complexes (Figure 5) were discussed based on the elemental analysis, conductance, FTIR, UV-Vis, ^1^HNMR, and TGA measurements. The various analyses performed in this study deduced that the coordination between the folic acid ligand and central metal ions passes through oxygen atoms of the carboxylate group and nitrogen atoms of amide groups.

### 3.8. XRD, SEM, and TEM Analyses

**Figure 6:**
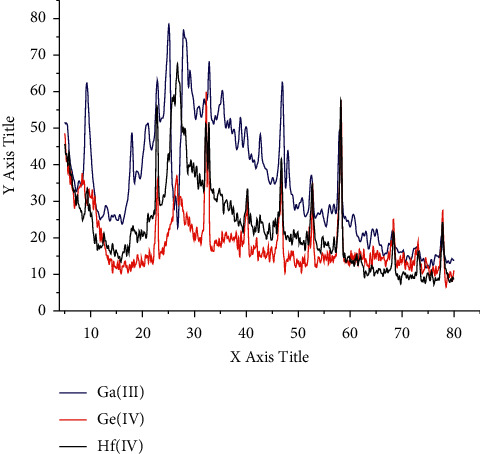
XRD patterns of Ga(III), Ge(IV), and Hf(IV) folate complexes.

**Figure 7:**
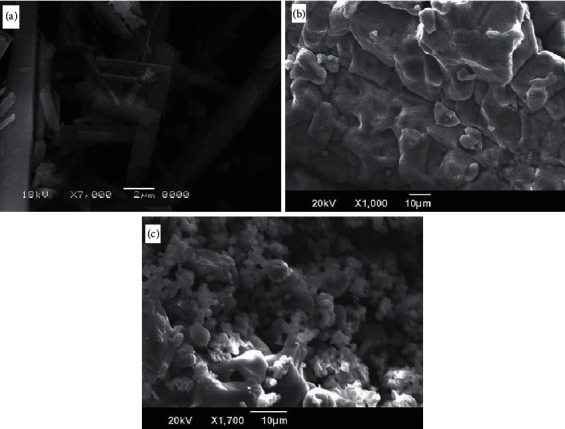
SEM images of (a) Ga(III), (b) Ge(IV), and (c) Hf(IV) folate complexes.

**Figure 8:**
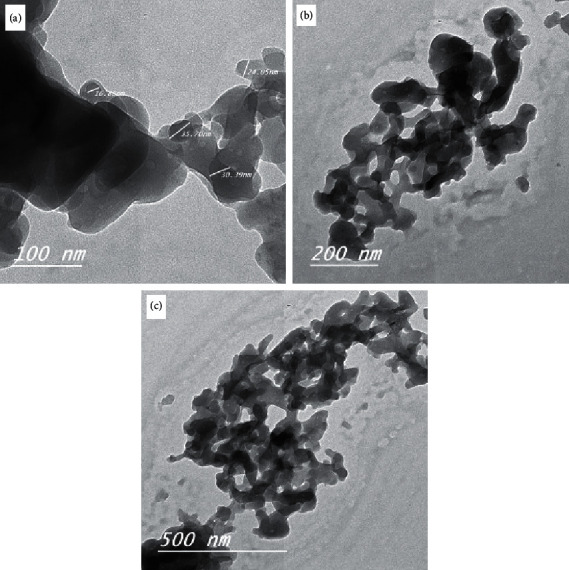
TEM pictures of (a) Ga(III), (b) Ge(IV), and (c) Hf(IV) folate complexes.

In X-ray diffractograms of the folate complexes, major patterns were scanned within the 4° to 80° 2*θ* range. The XRD patterns of the folate complexes (Figure 6) are completely different from those of folic acid [38] due to the formation of new coordination compounds. The X-ray diffractogram of the three synthesized metal-folate complexes is semicrystalline as well as amorphous in nature. According to the Scherrer equation, the crystallite sizes of the synthesized folate complexes were calculated using full width at half maximum of the diffraction peak. The crystallite sizes of the folate complexes were located at ∼30–100 nm. The SEM images of the synthesized complexes are given in Figure 7. The surface morphology changes with changes in the ionic radius of specific metal ions; both the images have many irregular shapes. TEM micrographs of the Ga^3+^, Ge^4+^, and Hf^4+^ complexes (Figure 8) demonstrated particles within a nanosize range. The average particle diameter for folate complexes was in the range of 30–100 nm, in agreement with the XRD data.

### 3.9. Biological Results

**Figure 9:**
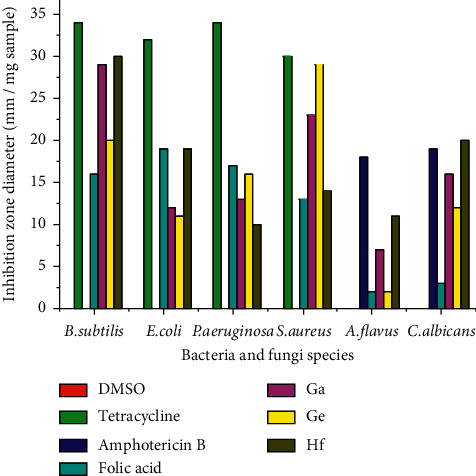
Inhibition zone diameter of DMSO control, “tetracycline” antibacterial agent, “amphotericin B” antifungal agent, and folic acid and its synthesized complexes against some bacteria and fungi species.

We report the results of in vitro microbial tests of the FAH_2_ drug and its gallium(III), germanium(IV), and hafnium(IV) complexes against a panel of bacteria and fungi species (Table 6 and Figure 9):

**Table:** 

*E. coli*	*B. subtilis*	*Aspergillus flavus*
*P. aeruginosa*	*S. aureus*	*Candida albicans*

Gallium(III), germanium(IV), and hafnium(IV) folate complexes were more active against *B. subtilis* and *S. aureus* bacteria than the free folic acid ligand drug. The hafnium(IV) complex has a bactericidal efficiency against *Escherichia coli.* All folate metal complexes have antifungal activity against different kinds of fungi understudy rather than free FAH_2_-free drugs. The folic acid ligand and its metal complexes displayed antibacterial and antifungal activities against the tested organisms. According to Tweedy's chelation theory [39], these data support the notion that metal ions enhance the antibacterial and antifungal activities by increasing lipophilicity, thus facilitating the penetration of metal complex across the cell membrane [39,40]. We tested the inhibitory concentration 50% (IC_50_) of the germanium(IV) complex with human hepatocellular carcinoma (HepG-2) cells (Table 7). The IC_50_ was higher than 1000 *µ*g/mL, indicating that the germanium(IV) complex has a significant efficiency against the HepG-2 cell line.

## 4. Conclusions

The metal chelation between Ga(III), Ge(IV), and Hf(IV) metal ions with folic acid with 1 : 2 molar ratio was prepared. The folic acid acts as a tridentate chelate via oxygen and nitrogen atoms of carboxylate and amido groups. The formulas of the synthesized folate complexes were NH_4_[Ga(FA)_2_]·4H_2_O, [Ge(FA)_2_]·3H_2_O, and [Hf(FA)_2_]·3H_2_O. These complexes have been discussed and assigned according to the microanalytical, conductance, FTIR, UV-Vis, ^1^HNMR, and TGA analyses. The thermal stability behavior of the synthesized folate complexes was confirmed as dependent on the calculation of kinetic thermodynamic parameters. The biological efficiency of the synthesized folate complexes was screened against bacteria and fungi species with significant values. The experimental IC_50_ data of the germanium(IV) complex in vitro showed the readiness of the Ge(IV) complex to be used as an antihepatocellular anticancer drug.

## Figures and Tables

**Table 1:** Infrared spectral frequency data of FAH_2_ and synthesized complexes.

Assignments	FAH_2_	Ga(III) complex	Ge(IV) complex	Hf(IV) complex
*ν* (OH); H_2_O	3558	3487	3486	3468
	3411			
*ν* (NH) amide	3318	3153	3158	3139
*ν*_as_ (CH)	3119	3043	3022	3003
	2933			
*ν*_s_ (CH)	2826	2810	2791	2790
Ν (COOH)	1702	1697	1697	1697
*ν*_as_ (COO−)	1604	1618	1618	1618
*δ* (NH) amide II	1564	1525	1523	1525
*ν*_s_ (COO−)	1485	1405	1401	1399
*ν*_as_ (CC)	1338	1286	1298	1259
*ν* (CN)	1192	1178	1178	1178
*ν*_s_ (CC)	913	970	900	966
*δ* (CC)	833	846	767	873
*ν* (M–O)	—	510	501	514

**Table 2:** Stretching vibration bands of the carboxylate group for the FAH_2_ and synthesized complexes.

Compound	*ν*_as_ (COO)	*ν*_s_ (COO)	∆ = *ν*_as_ (COO)−*ν*_s_ (COO)	Bonding mode
FAH_2_	1604	1485	119	—
Ga(III) complex	1618	1405	213	Monodentate
Ge(IV) complex	1618	1401	217	Monodentate
Hf(IV) complex	1610	1399	211	Monodentate

**Table 3:** The ^1^HNMR spectral data of the FAH_2_ and synthesized complexes.

Number of protons	*δ* (ppm)		
	FAH_2_	Ga(III) complex	Ge(III) complex
H_7_	8.648	8.621, 8.617	8.627
H_18_	8.093	7.788, 7.766	7.778, 7.755
H_13,15_	7.664	7.597	7.592
H_10_	7.635	7.571	7.563
H_12, 16_	6.630, 6.659	6.647, 6.621	6.648, 6.557
H_9_	4.496	4.464, 4.449	4.467, 4.447
H_19_	4.476	4.164, 4.144	4.154, 4.131
H_22_	2.504, 2.501, 2.495	2.507, 2.501, 2.495	2.513, 2.507, 2.501
H_21a_	2.317	2.224, 2.202	2.223, 2.1987
H_21b_	2.293	1.971, 1.950, 1.903	1.937, 1.916, 1.892

**Table 4:** TGA decomposition data of the FAH_2_ ligand and synthesized complexes.

Compounds	Steps	Temp. range (°C)	Decomposed assignments	Residual species	Mass loss (%)
Ga(III)	1^st^	56–181	−4H_2_O + NH_3_	Ga + carbon	90.698
	2^nd^	181–445	−7C_2_H_2_ + 2NO + 5N_2_		
	3^rd^	445–800	−10C_2_H_2_ + 2CO + 4O_2_ + N_2_		

Ge(IV)	1^st^	30–146	−3H_2_O	Ge + carbon	84.167
	2^nd^	146–321	−7C_2_H_2_ + 2NO + 5N_2_		
	3^rd^	321–800	−10C_2_H_2_ + 2CO + 4O_2_ + N_2_		

Hf(IV)	1^st^	34–154	−3H_2_O	Hf + carbon	84.023
	2^nd^	154–380	−7C_2_H_2_ + 2NO + 5N_2_		
	3^rd^	380–800	−10C_2_H_2_ + 2CO + 4O_2_ + N_2_		

**Table 5:** Kinetic thermodynamic parameters of the FAH_2_ and synthesized complexes.

Compound	Steps	Coats–Redfern equation				
		*r*	*E* (kJ mol^−1^)	∆*S* ^*∗*^ (Jk^−1^mol^−1^)	∆*H* ^*∗*^ (kJ mol^−1^)	∆*G* ^*∗*^ (kJ mol^−1^)
Ga(III) complex	1^st^	0.9976	4.95 ^*∗*^10^4^	−1.68 ^*∗*^10^2^	4.63 ^*∗*^10^4^	1.12 ^*∗*^10^5^
	2^nd^	0.9429	2.82 ^*∗*^10^4^	−1.38 ^*∗*^10^2^	2.34 ^*∗*^10^4^	1.04 ^*∗*^10^5^
	3^rd^	0.9872	1.10 ^*∗*^10^5^	−1.79 ^*∗*^10^2^	1.02 ^*∗*^10^5^	2.62 ^*∗*^10^5^

Ge(IV) complex	1^st^	0.9896	2.91 ^*∗*^10^4^	−1.71 ^*∗*^10^2^	2.61 ^*∗*^10^4^	8.80 ^*∗*^10^4^
	2^nd^	0.9808	5.02 ^*∗*^10^4^	−1.75 ^*∗*^10^2^	4.60 ^*∗*^10^4^	1.34 ^*∗*^10^5^
	3^rd^	0.9477	5.21 ^*∗*^10^4^	−1.43 ^*∗*^10^2^	4.52 ^*∗*^10^4^	1.64 ^*∗*^10^5^

Hf(IV) complex	1^st^	0.9987	3.18 ^*∗*^10^4^	−1.65 ^*∗*^10^2^	2.87 ^*∗*^10^4^	8.94 ^*∗*^10^4^
	2^nd^	0.9892	4.30 ^*∗*^10^4^	−1.62 ^*∗*^10^2^	3.85 ^*∗*^10^4^	1.26 ^*∗*^10^5^
	3^rd^	0.9970	4.90 ^*∗*^10^4^	−1.60 ^*∗*^10^2^	4.45 ^*∗*^10^4^	1.32 ^*∗*^10^5^

Compound	Steps	Horowitz and Metzger equation				
		*r*	*E* (kJ mol^−1^)	∆*S* ^*∗*^ (Jk^−1^mol^−1^)	∆*H* ^*∗*^ (kJ mol^−1^)	∆*G* ^*∗*^ (kJ mol^−1^)
Ga(III) complex	1^st^	0.9895	5.60 ^*∗*^10^4^	−1.42 ^*∗*^10^2^	5.27 ^*∗*^10^4^	1.08 ^*∗*^10^5^
	2^nd^	0.9372	3.62 ^*∗*^10^4^	−2.37 ^*∗*^10^2^	3.13 ^*∗*^10^4^	1.70 ^*∗*^10^5^
	3^rd^	0.9858	1.27 ^*∗*^10^5^	−1.56 ^*∗*^10^2^	1.20 ^*∗*^10^5^	2.60 ^*∗*^10^5^

Ge(IV) complex	1^st^	0.9846	3.50 ^*∗*^10^4^	−1.90 ^*∗*^10^2^	3.20 ^*∗*^10^4^	1.01 ^*∗*^10^5^
	2^nd^	0.9969	5.94 ^*∗*^10^4^	−1.73 ^*∗*^10^2^	5.51 ^*∗*^10^4^	1.43 ^*∗*^10^5^
	3^rd^	0.9706	6.78 ^*∗*^10^4^	−2.21 ^*∗*^10^2^	6.09 ^*∗*^10^4^	2.45 ^*∗*^10^5^

Hf(IV) complex	1^st^	0.9778	3.95 ^*∗*^10^4^	−1.78 ^*∗*^10^2^	3.64 ^*∗*^10^4^	1.02 ^*∗*^10^5^
	2^nd^	0.9827	5.20 ^*∗*^10^4^	−1.97 ^*∗*^10^2^	4.75 ^*∗*^10^4^	1.54 ^*∗*^10^5^
	3^rd^	0.9822	6.80 ^*∗*^10^4^	−1.92 ^*∗*^10^2^	5.80 ^*∗*^10^4^	2.34 ^*∗*^10^5^

**Table 6:** Antimicrobial assessments of Ga(III), Ge(IV), and Hf(IV) folate complexes.

Sample		Inhibition zone diameter (mm/mg sample)					
		*Bacillus subtilis* (G^+^)	*Escherichia coli* (G^−^)	*Pseudomonas aeruginosa* (G^−^)	*Staphylococcus aureus* (G^+^)	*Aspergillus flavus* (fungus)	*Candida albicans* (fungus)
Control: DMSO		0.0	0.0	0.0	0.0	0.0	0.0
Standard	Tetracycline antibacterial agent	34	32	34	30	—	—
	Amphotericin B antifungal agent	—	—	—	—	18	19
FAH_2_		16	19	17	13	2	5
Ga(III) complex		29	12	13	23	7.0	16
Ge(IV) complex		20	11	16	29	2.0	12
Hf(IV) complex		30	19	10	14	11	20

G : gram reaction. Solvent: DMSO.

**Table 7:** The inhibitory activities of the Ge(IV) complex against HepG cell line.

Conc. (*µ*g/mL)	HepG cell line								SE
	Ab	% C	Ab	% C	Ab	% C	Abs av	Ave % C	
0	0.453	97.41935	0.476	102.3656	0.466	100.2151	0.465	100	1.431899
0.1	0.429	92.25806	0.413	88.8172	0.401	86.0515	0.414333	89.10394	1.79521
1	0.408	87.74194	0.400	86.02151	0.403	86.66667	0.403667	86.81004	0.501792
10	0.411	88.3871	0.390	83.87097	0.404	86.88172	0.401667	86.37993	1.327617
100	0.379	81.50538	0.384	82.58065	0.390	83.87097	0.384333	82.65233	0.683827
1000	0.254	54.62366	0.256	55.05376	0.25	53.76344	0.253333	54.48029	0.379319

## Data Availability

The data associated with this study can be accessed from the first author upon a reasonable request.

## Supplementary Materials

Supplementary Materials Supplementary files included in the manuscript are ^1^HNMR spectra of the FAH_2_-free ligand (Figure 1S), Ga(III) complex (Figure 2S), and Ge(IV) complex (Figure 3S) in DMSO-d_6_.
