# Supplementary material for: Novel Gallium(III), Germanium(IV), and Hafnium(IV) Folate Complexes and Their Spectroscopic, Thermal Decomposition, Morphological, and Biological Characteristics
Source: Bioinorg Chem Appl. 2020 Dec 18;2020:6678688. doi: 10.1155/2020/6678688 (PMC7769666; doi:10.1155/2020/6678688)

MoaminSalah-P19-DMSO-H1

Archive directory: /export/home/vnmr1/vnmrsys/data
Sample directory: DD5mm_test_12Mar2014-21:34:40
File: PROTON

Pulse Sequence: s2pul

Solvent: DMSO
Temp. 30.0 C / 303.1 K
Mercury-300BB "NMR300"

Relax. delay 1.000 sec
Pulse 45.0 degrees
Acq. time 4.853 sec
Width 6600.7 Hz
24 repetitions
OBSERVE H1, 300.0687871 MHz
DATA PROCESSING
FT size 65536
Total time 43 min, 34 sec
Date: Sep 21 2016

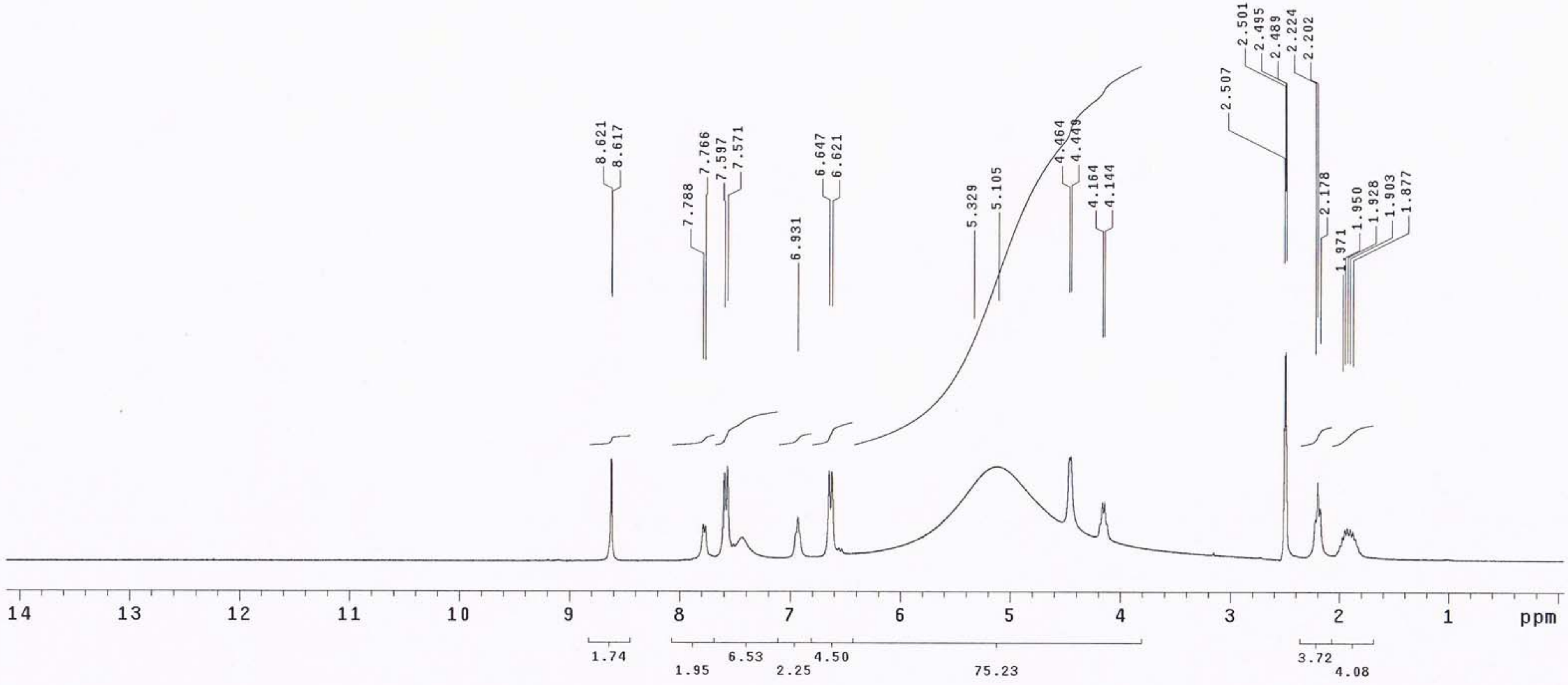

Supplement: Supplementary Materials — Supplementary files included in the manuscript are 1HNMR spectra of the FAH2-free ligand (Figure 1S), Ga(III) complex (Figure 2S), and Ge(IV) complex (Figure 3S) in DMSO-d6. [file 6678688.f1.zip › 6678688.f1/Fig 2S HNMR of Ga-complex.pdf]
